# Supplementary material for: Efficient generation of mNeonGreen Plasmodium falciparum reporter lines enables quantitative fitness analysis
Source: Front Cell Infect Microbiol. 2022 Sep 16;12:981432. doi: 10.3389/fcimb.2022.981432 (PMC9523114; doi:10.3389/fcimb.2022.981432)
Supplement: Supplementary file 2 [file DataSheet_1.pdf]

Supplementary Material

Supplemental Figure 1. Alignment of 5' and 3' homologous regions of the *pfpare* locus in *P. falciparum* sequences. The DNA sequences of the 5' (**A**) and 3' (**B**) *pfpare* homologous regions used in pDC2-coCas9-*pare*-BSD-mNeonGreen were obtained for *P. falciparum* 3D7, HB3, 7G8, GB4, CD01, GA01, IT and Dd2 strains from PlasmoDB (Amos et al., 2021). Clustal Omega was used to create a multiple sequence alignment (Sievers et al., 2011, Goujon et al., 2010). Single nucleotide polymorphisms are highlighted with yellow boxes.

A

```
3D7  ATGAAGAGCCAGGGTGGAGGGAAGATATCGAGGAAGAGCTCCACAGGTTCGAGCAGTAGTCGATTAGATGGGAATCCCAAATTGGATTCT 90
HB3  ATGAAGAGCCAGGGTGGAGGGAAGATATCGAGGAAGAGCTCCACAGGTTCGAGCAGTAGTCGATTAGATGGGAATCCCAAATTGGATTCT 90
7G8  ATGAAGAGCCAGGGTGGAGGGAAGATATCGAGGAAGAGCTCCACAGGTTCGAGCAGTAGTCGATTAGATGGGAATCCCAAATTGGATTCT 90
GB4  ATGAAGAGCCAGGGTGGAGGGAAGATATCGAGGAAGAGCTCCACAGGTTCGAGCAGTAGTCGATTAGATGGGAATCCCAAATTGGATTCT 90
CD01 ATGAAGAGCCAGGGTGGAGGGAAGATATCGAGGAAGAGCTCCACAGGTTCGAGCAGTAGTCGATTAGATGGGAATCCCAAATTGGATTCT 90
GA01 ATGAAGAGCCAGGGTGGAGGGAAGATATCGAGGAAGAGCTCCACAGGTTCGAGCAGTAGTCGATTAGATGGGAATCCCAAATTGGATTCT 90
IT   ATGAAGAGCCAGGGTGGAGGGAAGATATCGAGGAAGAGCTCCACAGGTTCGAGCAGTAGTCGATTAGATGGGAATCCCAAATTGGATTCT 90
Dd2  ATGAAGAGCCAGGGTGGAGGGAAGATATCGAGGAAGAGCTCCACAGGTTCGAGCAGTAGTCGATTAGATGGGAATCCCAAATTGGATTCT 90
     ******************************************************************************************

3D7  TTTCATAATAAGGATGGGTTATCTTTAAAAACTTATGCATGGACGGTTAAAAATCCAGTAGGTGTTATAATAGCATGTCATGGTATGAAT 180
HB3  TTTCATAATAAGGATGGGTTATCTTTAAAAACTTATGCATGGACGGTTAAAAATCCAGTAGGTGTTATAATAGCATGTCATGGTATGAAT 180
7G8  TTTCATAATAAGGATGGGTTATCTTTAAAAACTTATGCATGGACGGTTAAAAATCCAGTAGGTGTTATAATAGCATGTCATGGTATGAAT 180
GB4  TTTCATAATAAGGATGGGTTATCTTTAAAAACTTATGCATGGACGGTTAAAAATCCAGTAGGTGTTATAATAGCATGTCATGGTATGAAT 180
CD01 TTTCATAATAAGGATGGGTTATCTTTAAAAACTTATGCATGGACGGTTAAAAATCCAGTAGGTGTTATAATAGCATGTCATGGTATGAAT 180
GA01 TTTCATAATAAGGATGGGTTATCTTTAAAAACTTATGCATGGACGGTTAAAAATCCAGTAGGTGTTATAATAGCATGTCATGGTATGAAT 180
IT   TTTCATAATAAGGATGGGTTATCTTTAAAAACTTATGCATGGACGGTTAAAAATCCAGTAGGTGTTATAATAGCATGTCATGGTATGAAT 180
Dd2  TTTCATAATAAGGATGGGTTATCTTTAAAAACTTATGCATGGACGGTTAAAAATCCAGTAGGTGTTATAATAGCATGTCATGGTATGAAT 180
     ******************************************************************************************

3D7  TCTCATGTACGTTTAGAATATTTAAGACATAATGTCGAGGTAGTAAATAATAATAAGGCAATATTAAAAGATGGT 255
HB3  TCTCATGTACGTTTAGAATATTTAAGACATAATGTCGAGGTAGTAAATAATAATAAGGCAATATTAAAAGATGGT 255
7G8  TCTCATGTACGTTTAGAATATTTAAGACATAATGTCGAGGTAGTAAATAATAATAAGGCAATATTAAAAGATGGT 255
GB4  TCTCATGTACGTTTAGAATATTTAAGACATAATGTCGAGGTAGTAAATAATAATAAGGCAATATTAAAAGATGGT 255
CD01 TCTCATGTACGTTTAGAATATTTAAGACATAATGTCGAGGTAGTAAATAATAATAAGGCAATATTAAAAGATGGT 255
GA01 TCTCATGTACGTTTAGAATATTTAAGACATAATGTCGAGGTAGTAAATAATAATAAGGCAATATTAAAAGATGGT 255
IT   TCTCATGTACGTTTAGAATATTTAAGACATAATGTCGAGGTAGTAAATAATAATAAGGCAATATTAAAAGATGGT 255
Dd2  TCTCATGTACGTTTAGAATATTTAAGACATAATGTCGAGGTAGTAAATAATAATAAGGCAATATTAAAAGATGGT 255
     ***************************************************************************
```

B

```
3D7  TATGATATCTATAGATGAGTTAGCAACGAAACCATCATATAAATATTTCTATATTCCATTAGCTAAATTCTTAGGAAGCTTTTTTCCAAG 725
HB3  TATGATATCTATAGATGAGTTAGCAACGAAACCATCATATAAATATTTCTATATTCCATTAGCTAAATTCTTAGGAATCTTTTTTCCAAG 725
7G8  TATGATATCTATAGATGAGTTAGCAACGAAACCATCATATAAATATTTCTATATTCCATTAGCTAAATTCTTAGGAATCTTTTTTCCAAG 725
GB4  TATGATATCTATAGATGAGTTAGCAACGAAACCATCATATAAATATTTCTATATTCCATTAGCTAAATTCTTAGGAAGCTTTTTTCCAAG 725
CD01 TATGATATCTATAGATGAGTTAGCAACGAAACCATCATATAAATATTTCTATATTCCATTAGCTAAATTCTTAGGAAGCTTTTTTCCAAG 725
GA01 TATGATATCTATAGATGAGTTAGCAACGAAACCATCATATAAATATTTCTATATTCCATTAGCTAAATTCTTAGGAAGCTTTTTTCCAAG 725
IT   TATGATATCTATAGATGAGTTAGCAACGAAACCATCATATAAATATTTCTATATTCCATTAGCAAAATTCTTAGGAAGCTTTTTTCCAAG 725
Dd2  TATGATATCTATAGATGAGTTAGCAACGAAACCATCATATAAATATTTCTATATTCCATTAGCAAAATTCTTAGGAAGCTTTTTTCCAAG 725
     *************************************************************** ************* ************

3D7  TTTACGTCTTACTCCTGGTTTACGTTTTAATATGTTTCCACATATGAATGATATTATGGAATTTGATAAATTCAAATTTAAAAAACATGT 815
HB3  TTTACGTCTTACTCCTGGTTTACGTTTTAATATGTTTCCACATATGAATGATATTATAGAATTTGATAAATTCAAATTTAAAAAACATGT 815
7G8  TTTACGTCTTACTCCTGGTTTACGTTTTAATATGTTTCCACATATGAATGATATTATAGAATTTGATAAATTCAAATTTAAAAAACATGT 815
GB4  TTTACGTCTTACTCCTGGTTTACGTTTTAATATGTTTCCACATATGAATGATATTATAGAATTTGATAAATTCAAATTTAAAAAACATGT 815
CD01 TTTACGTCTTACTCCTGGTTTACGTTTTAATATGTTTCCACATATGAATGATATTATAGAATTTGATAAATTCAAATTTAAAAAACATGT 815
GA01 TTTACGTCTTACTCCTGGTTTACGTTTTAATATGTTTCCACATATGAATGATATTATAGAATTTGATAAATTCAAATTTAAAAAACATGT 815
IT   TTTACGTCTTACTCCTGGTTTACGTTTTAATATGTTTCCACATATGAATGATATTATAGAATTTGATAAATTCAAATTTAAAAAACATGT 815
Dd2  TTTACGTCTTACTCCTGGTTTACGTTTTAATATGTTTCCACATATGAATGATATTATAGAATTTGATAAATTCAAATTTAAAAAACATGT 815
     ********************************************************* ********************************

3D7  AACATGTAGATTAGGTTATGAGTTATTAAATGCTATAAATAACCTAAATAATGATATGGATTACATTCCTGAAAATACACCTATACTTTT 905
HB3  AACATGTAGATTAGGTTATGAGTTATTAAATGCTATAAATAACCTAAATAATGATATGGATTACATTCCTGAAAATACACCTATACTTTT 905
7G8  AACATGTAGATTAGGTTATGAGTTATTAAATGCTATAAATAACCTAAATAATGATATGGATTACATTCCTGAAAATACACCTATACTTTT 905
GB4  AACATGTAGATTAGGTTATGAGTTATTAAATGCTATAAATAACCTAAATAATGATATGGATTACATTCCTGAAAATACACCTATACTTTT 905
CD01 AACATGTAGATTAGGTTATGAGTTATTAAATGCTATAAATAACCTAAATAATGATATGGATTACATTCCTGAAAATACACCTATACTTTT 905
GA01 AACATGTAGATTAGGTTATGAGTTATTAAATGCTATAAATAACCTAAATAATGATATGGATTACATTCCTGAAAATACACCTATACTTTT 905
IT   AACATGTAGATTGGGTTATGAGTTATTAAATGCTATAAATAACCTAAATAATGATATGGATTACATTCCTGAAAATACACCTATACTTTT 905
Dd2  AACATGTAGATTGGGTTATGAGTTATTAAATGCTATAAATAACCTAAATAATGATATGGATTACATTCCTGAAAATACACCTATACTTTT 905
     ************ *****************************************************************************

3D7  TGCTCACTCAAAAAAAGATAGTGTATGCTTTTATGGAGGTACATTAAAATTTTACAACAAACTTAAGTGTCTTAAAAAAGAATTATATAC 995
HB3  TGCTCACTCAAAAAAAGATAGTGTATGCTTTTATGGAGGTACATTAAAATTTTACAACAAACTTAAGTGTCTTAAAAAAGAATTATATAC 995
7G8  TGCTCACTCAAAAAAAGATAGTGTATGCTTTTATGGAGGTACATTAAAATTTTACAACAAACTTAAGTGTCTTAAAAAAGAATTATATAC 995
GB4  TGCTCACTCAAAAAAAGATAGTGTATGCTTTTATGGAGGTACATTAAAATTTTACAACAAACTTAAGTGTCTTAAAAAAGAATTATATAC 995
CD01 TGCTCACTCAAAAAAAGATAGTGTATGCTTTTATGGAGGTACATTAAAATTTTACAACAAACTTAAGTGTCTTAAAAAAGAATTATATAC 995
GA01 TGCTCACTCAAAAAAAGATAGTGTATGCTTTTATGGAGGTACATTAAAATTTTACAACAAACTTAAGTGTCTTAAAAAAGAATTATATAC 995
IT   TGCTCACTCAAAAAAAGATAGTGTATGCTTTTATGGAGGTACATTAAAATTTTACAACAAACTTAAGTGTCTTAAAAAAGAATTATATAC 995
Dd2  TGCTCACTCAAAAAAAGATAGTGTATGCTTTTATGGAGGTACATTAAAATTTTACAACAAACTTAAGTGTCTTAAAAAAGAATTATATAC 995
     ******************************************************************************************

3D7  CTTAGATGACATGGACCACCTTCTACCTATGGAACCTGGAAATGAAAGAGTTCTAAAAAAAATTATCACATGGCTAGCTGTCCATACCCC 1085
HB3  CTTAGATGACATGGACCACCTTCTACCTATGGAACCTGGAAATGAAAGAGTTCTAAAAAAAATTATCACATGGCTAGCTGTCCATACCCC 1085
7G8  CTTAGATGACATGGACCACCTTCTACCTATGGAACCTGGAAATGAAAGAGTTCTAAAAAAAATTATCACATGGCTAGCTGTCCATACCCC 1085
GB4  CTTAGATGACATGGACCACCTTCTACCTATGGAACCTGGAAATGAAAGAATTCTAAAAAAAATTATCACATGGCTAGCTGTCCATACCCC 1085
CD01 CTTAGATGACATGGACCACCTTCTACCTATGGAACCTGGAAATGAAAGAATTCTAAAAAAAATTATCACATGGCTAGCTGTCCATACCCC 1085
GA01 CTTAGATGACATGGACCACCTTCTACCTATGGAACCTGGAAATGAAAGAATTCTAAAAAAAATTATCACATGGCTAGCTGTCCATACCCC 1085
IT   CTTAGATGACATGGACCACCTTCTACCTATGGAACCTGGAAATGAAAGAGTTCTAAAAAAAATTATCACATGGCTAGCTGTCCATACCCC 1085
Dd2  CTTAGATGACATGGACCACCTTCTACCTATGGAACCTGGAAATGAAAGAGTTCTAAAAAAAATTATCACATGGCTAGCTGTCCATACCCC 1085
     ************************************************* ****************************************

3D7  CAAACAAGAAGAACAA 1101
HB3  CAAACAAGAAGAACAA 1101
7G8  CAAACAAGAAGAACAA 1101
GB4  CAAACAAGAAGAACAA 1101
CD01 CAAACAAGAAGAACAA 1101
GA01 CAAACAAGAAGAACAA 1101
IT   CAAACAAGAAGAACAA 1101
Dd2  CAAACAAGAAGAACAA 1101
     ****************
```
